# Ramadhan fasting for people living with chronic illness: A narrative literature review

**DOI:** 10.4102/safp.v66i1.5805

**Published:** 2024-01-31

**Authors:** Tasleem Ras, Rashiqua Holdman, Dianne Matthews

**Affiliations:** 1 Department of Family, Community and Emergency Care, Faculty of Health Sciences, University of Cape Town, Cape Town, South Africa

**Keywords:** fasting, Ramadhan, non-communicable diseases, cultural sensitivity, mental health, geriatrics

## Abstract

Muslims constitute approximately 20% of the world’s population. In South Africa, Muslims constitute just under 2% of the total population. Fasting is one of the mandatory activities of adherents of the Islamic faith, where all healthy adult Muslims abstain from food, drink, and sexual activities between dawn and dusk during the month of Ramadhan. Medical doctors are frequently required to provide advice to their Muslim patients about the safety or other health impacts of this type of fasting. This narrative review provides an overview of research conducted on Muslim populations during the fasting period, with special reference to non-communicable diseases (NCDs) that are prevalent in the Muslim community. In the absence of evidence-based clinical guidelines, this article summarises the latest published research on this topic, providing a resource for clinicians and researchers. This paper provides an evidence summary to clinicians when engaging with their patients who may be engaging in Ramadhan fasting, while also identifying gaps in the body of evidence that could inform future research.

## Introduction

Muslims, people who adhere to the Islamic faith, constitute approximately two billion of the world’s eight billion people.^1^ In South Africa, estimates put the number of Muslims at about 900 000, though this is an unverified amount.^2^ One of the central practices of Islam is the observance of fasting during Ramadhan, the ninth month of the Islamic lunar calendar.

During this time, healthy Muslim adults are required to abstain from food, beverages, smoking and spousal conjugal relations between dawn and sunset. This practice is repeated daily for the duration of the month, which lasts 29 days or 30 days. Breastfeeding mothers, pregnant or menstruating women, those suffering from illness, the frail elderly unable to tolerate fasting, and travellers are exempt from the responsibility. In this case, they may either repeat the fast later as appropriate or pay *fidyah* [feed the poor] if permanently unable to fast.

The South African population, including Muslims, are faced with the quadruple burden of disease of communicable and non-communicable diseases (NCDs), maternal-child morbidity and mortality and violence. Non-communicable diseases that have a metabolic component can be impacted by fasting, and cognisance must be paid to the potential risk, or benefits, this poses to the fasting person. This article provides a narrative from the literature on the effects of fasting on some NCDs commonly found in the South African population.

## Method

We conducted a literature search of the online databases PubMed, SABINET and Scielo. Additionally, we searched Google Scholar. The keywords used were ‘Ramadhan fasting’ (alternative spellings were ‘Ramadhan’ and ‘Ramadan’) and ‘health impact’ or ‘health’ or ‘clinical guidelines’ or ‘mental health’ or ‘elderly’ or ‘physiology’ or ‘diabetes mellitus’ or ‘metabolic disease’. We did not insert a time limit to these searches, given the relative paucity of the literature. The searches generated a list of titles that we reviewed and included those which met our inclusion criteria. The criteria we used were that the article needed to focus specifically on Ramadhan fasting (as opposed to intermittent or other types of fasting) and addressed the key disease areas we were focussing on. For those titles that we included, we reviewed the abstract to ensure that it addressed the specific content for the relevant section that we had determined *a priori*. Finally, those studies that were included to inform this paper were read iteratively, and their methodology was appraised to ensure that the findings could be included in this review. Because we were not attempting to do a scoping or systematic review, it was not deemed necessary to keep track of the number of articles our searches generated, or their flow as we progressively applied our criteria.

## Physiology of Ramadhan fasting

The key physiological changes in Ramadhan fasting occur because of the changed diet with a total fasting period of between 13 h and 16 h (depending on the country and the season that Ramadhan falls into). The alteration in diet is related to the changed frequency of eating, with lunch being excluded altogether. Additionally, while not documented in the local literature, anecdotal experiences indicate that the quality of food, largely culturally driven, changes towards a carbohydrate-rich diet with increased caloric intake. Extra night-time prayers that alter sleeping patterns are also prevalent.

The lack of water intake during daylight hours has not been associated with persistent impaired renal function or significant electrolyte imbalances in healthy individuals.^3^ A decrease in 24-h urine output and an increase in urine osmolality have been associated with fasting in various climates, though this usually corrects once the fasting period is over.^3^

In the short-term, the data suggests that this type of fasting has beneficial effects on glucose and lipid metabolism. A meta-analysis of glucose levels in fasting populations indicated clinically small, but statistically significant decreases in fasting glucose levels after Ramadhan fasting.^4,5,6^ The same meta-analysis found that high-density lipoprotein (HDL-C) was increased after fasting, while total cholesterol and low-density lipoproteins (LDL-C) levels were decreased. Additionally, total body weight decreased, though this weight loss was not sustained in the subsequent months.^7^

Sleep pattern alterations have been reported in studies involving populations in Muslim-majority countries, where social and spiritual activities are enacted at night during this month. Disrupted circadian rhythms have been shown to directly impact cortisol levels, which in turn affect insulin secretion and action, and hence glucose control.^8^

## Diabetes mellitus

The metabolic link between Ramadhan fasting and diabetes mellitus is well documented, with several evidence-based guidelines published internationally.^9,10,11^ For those people living with diabetes who choose to fast, there need to be a high level of awareness regarding the risk for hyperglycaemia, hypoglycaemia, complications of dehydration, and concomitant hyperosmolar coma or ketoacidosis.^9^

**FIGURE 1:**
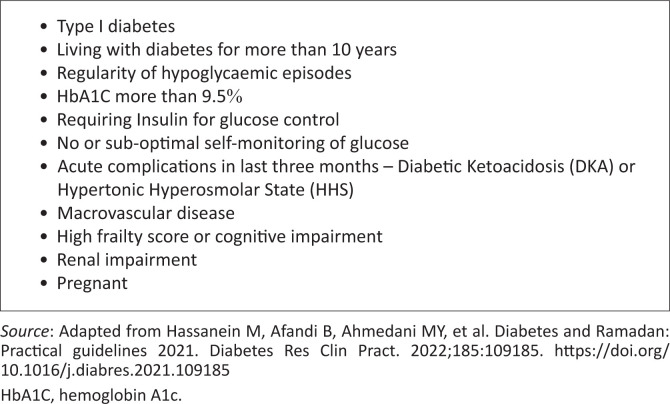
Independent risk factors for adverse outcomes.

A risk stratification for fasting-related adverse events has been developed by the International Diabetes Federation-Diabetes and Ramadhan (IDF-DaR) writing group, categorising patients into high, moderate and low risk.^9^ According to this system, the key independent risk factors for adverse outcomes are listed in Figure 1. The algorithm scores patients as low-risk if their cumulative score is less than 3, moderate if between 3 and 6, and high if more than 6. In practical terms, this means that if any target organ damage is present, or if two other factors are present, patients are placed in the high-risk category.

Based on this stratification, patients can be advised that it is safe to fast (low-risk), that fasting may be attempted with close monitoring (moderate-risk), or that fasting should not be attempted at all (high-risk). It is important to note that this system has not yet been tested in controlled trials and is based on expert experiences and observational studies.

As with any chronic disease, patient education is key to long term success and complication prevention. Key elements of a pre-Ramadhan patient education programme should be general diabetes education, especially focussing on metabolic control and risk awareness, self-monitoring of glucose, and lifestyle advice on diet, hydration and activity levels.^12^ A word of caution to the healthcare provider: social practices in Ramadhan are steeped in cultural tradition, and when counselling for behaviour change, a high level of cultural sensitivity and humility needs to be demonstrated.^13^

The IDF-DaR guideline^9^ suggests the following when assisting people living with diabetes in managing their condition:

- A pre-Ramadhan visit about 6–8 weeks before the months starts. At this visit, knowledge-sharing, risk assessment, and therapeutic optimisation and adjustment are performed, allowing patients to make informed decisions about fasting. Clear advice should be given about recognising complications and when fasting should not be continued.
- Self-monitoring by patient during the fasting period, with close availability of the healthcare professional for advice and consultation.
- A post-Ramadhan assessment, with a focus on fasting experiences and customised to the risk category.

The most common complication of fasting in people living with diabetes is hypoglycaemia, especially if patients are using insulin regimens or sulfonylureas, although the newer sulfonylureas like glimepiride and gliclazide tend to have a better safety profile than the older drugs.^9^ These medications will need dose adjustments at the pre-Ramadhan visit. There is consensus that Metformin does not need dose adjustment for fasting, though an 8 hourly regime may need to be modified to a twice daily regime. Preliminary evidence indicates that the newer anti-diabetic medications are safe to use in the fasting state.^14^ However, an important caveat is that many of these patients on multiple anti-diabetic medications tend to be elderly, and so may have other co-morbidities or metabolic dysregulation that may affect their risk status.

## Hypertension

Hypertension is probably the most common NCD affecting long-term cardiac and renal health in South Africa, with an estimated prevalence of 30.4%.^15^ In a systematic review, Ramadhan fasting was shown to have a lowering effect on blood pressure control in healthy individuals, and in those with established hypertension.^16^ Additionally, fasting was found to have varied effect on systolic and diastolic blood pressures.^17^ These two systematic reviews were unable to do a meta-analysis because of the high level of heterogeneity in their studies, but nonetheless indicate that fasting is safe for those patients living with hypertension, and in some instances, even beneficial, with the beneficial effect lasting a variable period after the fasting month had concluded. Another systematic review that was able to complete a meta-analysis indicated that blood pressure control was improved for fasting people when they had hypertension, had hypertension with co-morbid diabetes, but not if they had co-morbid renal impairment.^18^

Despite the potential short-term benefits that accrue to the fasting person with hypertension, the evidence is weak when considering the long-term benefits on cardiovascular health, and consequently, there can be no claims to long-term cardio-protection.^19^ We could find no reports of adverse effects of anti-hypertensive medications in individuals who were fasting. This was surprisingly true even when diuretics were assessed during the fasting period, though the single observational study that evaluated and reported on this had a relatively small sample size.^20^ Additionally, another observational study in Qatar indicated no difference in stroke prevalence before, during, and after Ramadhan.^21^

## Cancer

Cancer prevalence in South Africa has shown a significant increase from 2001, when the National Cancer Registry showed a total number of 59 213 cancer diagnoses and in 2020, a total number of 76 449 diagnoses.^22^ These patients may undergo a variety of surgical, radiation or chemotherapeutic interventions. Given the life-threatening nature of cancer, many patients would opt to increase their spiritual activities, including Ramadhan fasting.

While the literature indicates that different types of fasting (ketogenic and calory restricted) could have limiting effects on cancer growth and improved chemotherapy pharmacokinetics, there is little to be found on Ramadhan fasting or intermittent fasting, which has similar physiological effects, except that water intake is allowed during intermittent fasting.^23^ The phases of the cancer journey include the process to a definitive diagnosis, implementing treatment modalities and the recovery phase. Key physiological challenges that emerge from these phases are the optimisation and maintenance of hydration, caloric intake, and protein levels. Patients have variably opted to fast during the month, with a Turkish study indicating that stage of disease was independently associated with the ability to maintain the fast, with advanced stage linked to lower likelihood of fasting.^24^ Islamic scholars are unanimous in their opinion that fasting is not mandatory for the cancer patient, with strong recommendations not to fast in advanced or symptomatic disease.^25^

## Renal disease

Chronic renal disease, described as an estimated glomerular filtration rate (eGFR) less than 60 mL/min, and chiefly resulting from poor diabetes mellitus or hypertension control, can be impacted by fasting, with the chief mechanism being short-term dehydration causing acute renal tubular cell damage.^26^ Though this finding is from an observational study, in the absence of higher levels of evidence it remains valid. Two clear recommendations emerge from this finding:

- Patients with impaired renal function should be counselled that fasting may be deleterious to their health.
- For those patients who opt to fast, evening hydration is mandatory.

Clinicians report anecdotally that renal colic because of renal stones is also fairly commonly encountered among fasting patients during the month of Ramadhan, with the hypothesis being increased stone formation during a period of relative dehydration. The literature is ambiguous about whether this holds true or not, with varying reports on increased incidence during this time.^26^ Interestingly, while an overall increased incidence was not reported, one study showed an increase in the first 2 weeks of Ramadhan, postulating that this was because of the sudden change in dietary pattern.^26^

A third element of renal disease relates to patients who have undergone renal transplant surgery. Observational studies indicate that where patients with stable renal function have fasted, no demonstrable renal damage could be found. The clinical recommendation for these patients is that fasting is safe if renal function has been stabilised for at least the preceding 6 months.^26^

## Mental health

Nearly one in three South Africans will suffer from a mental disorder in his or her lifetime, a higher prevalence than many low- and middle-income countries.^27^ Depression is a common mental illness, affecting approximately 15% – 20% of South Africans and is defined by the Diagnostic and Statistical Manual of Mental Disorders, Fifth Edition (DSM-V) as low mood or anhedonia with irritability, sleep disturbance, fatigue, motor function worsening, appetite or weight changes, poor concentration, memory and cognitive function impairment. In addition, guilt and suicidal thoughts are added features. These symptoms are not explained by other illnesses or substance use, and affect daily functionality.

Despite the use of anti-depressants as the mainstay of treatment for depression, it is only successful in approximately half of patients and induces multiple side effects.^28^ Hence, of particular interest in current research is the discovery of new pathophysiological pathways to develop more personalised treatments for depression and anxiety such as the gut–brain axis. The field of psycho–nutrition has developed extensively in recent years.^28^ Consequently, researchers have begun to explore the effects of different fasting strategies on the body to find dietary intervention programmes that are suitable for controlling obesity and which may have a positive impact on stress, anxiety, and depression.

Berthelot et al. found that post-Ramadhan scores for stress, anxiety and depression were lower compared to those before Ramadhan.^29^ They found that fasting groups had lower anxiety and depression levels compared to control groups when limiting the analyses to randomised controlled trials. Fasting was associated with decreased body mass index (BMI) in all studies without increased fatigue in fasting groups compared to controls. Adverse events were only reported for 1 day/week fasting.^28^ Other studies have shown that short-term fasting can increase negative emotions (depression, anxiety, anger, irritability, fatigue, and tension) and decrease positive emotions and vitality, especially during the first week of Ramadhan. This is contrasted with measurements from the third week onwards that show mood enhancement, which is reflected by increased positive mood and decreased negative mood.^28^ There are several factors which account for these contrasting results.

Firstly, strong religious beliefs can lead to positive effects on human physical and psychological health; for those who value their religious beliefs, fasting can be a pleasant and tolerant experience. In contrast, fasting may bring negative emotions to those who do not have religious beliefs. Secondly, fasting is closely related to self-emotional control. On the one hand, fasting is a process that requires considerable cognitive effort, including self-emotional control and successfully completing the fasting period may increase the feeling of self-control. Thirdly, different assessment tools for psychological targets may lead to different outcomes. Fourthly, previous fasting experience (whether this was a positive or negative experience) may influence the current fasting experience, resulting in different outcomes being reported. A study showed that where participants had never fasted before, the chances of low mood, irritability and other markers of mental health were worse.^29^ The same study found that participants with higher baseline levels of mental health indicators had greater effects at the end of the fasting period.^29^ Therefore, Ramadhan may have a positive effect on people who are already experiencing depression, anxiety, and stress.^30^

However, in patients with bipolar mood disorder or schizophrenia, the rates of acute relapse have been shown to be significantly higher when Ramadhan fasting is attempted.^31,32^ The recommendation for these groups of patients is that Ramadhan fasting should not be attempted.

## The elderly

In 2022, the national percentage of citizens aged 60 years and older in South Africa showed a steady increase to 9.2%, from 8.7% in 2017.^33^ This reflects an ageing population trend, with implications for healthcare delivery responsive to the needs of this population cohort. The elderly, defined as persons aged 60 years and older, represent a significant vulnerable group in respect of Ramadhan fasting.

An ageing physiology, increased chronic disease prevalence and polypharmacy, coupled with an ageing personality, present numerous clinical challenges in this group. Many primary care practitioners may recall a ‘difficult’ clinical encounter in which an elderly patient resolutely defends their right to fast during Ramadhan, perhaps against their clinical ‘best interests’. With this patient cohort, mindfulness of clinical risk factors and psycho-social health determinants by both the patient and healthcare provider, can contribute to optimal health outcomes during and post-Ramadhan.

Notable physiological alterations in the elderly include decreased cardiac output and raised blood pressure, progressively impaired glycaemic control, decreased creatinine clearance, slower cognitive processing, decreased neuronal adaptability to metabolic derangements, decreased muscle mass and increased degenerative joint conditions which, in combination with neuro-cognitive alterations, contribute to falls and fall-related injury.^34^ Even in the absence of co-morbidities, Ramadhan fasting may pose metabolic stress to the ageing physiology, if undertaken without nutritional and behavioural mindfulness.^35,36^

The literature provides some firm evidence of the impact of Ramadhan fasting on the elderly. Baccouche et al. demonstrated that a beneficial effect of Ramadhan fasting in elderly patients with cardiovascular risk factors was observed in the early post-fasting period, including increased HDL-C levels and better glycaemic control.^35^ In contrast, the study demonstrated a risk of renal function decrease with a simultaneous increase of glycaemia during the fasting period, which could be risky for diabetic patients.^35^ Hypertension and diabetes were found to be the commonest co-morbidities in fasting persons older than 60 years, across numerous studies.^37^ A longitudinal study on 182 fasting subjects aged 60 years and above, found a mild improvement in functional status and somatic complaints (though not statistically significant) with statistically significant improvements in depression, anxiety, insomnia, and social interaction before and after Ramadhan fasting (*p* = 0.001, *p* = 0.001, *p* = 0.006 and *p* = 0.002, respectively).^37^ Within this same study, statistically significant improvements in mean systolic and diastolic blood pressure were recorded in elderly subjects after fasting (*p* = 0.004 and *p* = 0.04, respectively).^37^

The effects of Ramadhan fasting on postural balance and attentional capacity has also been documented.^37^ In this interesting study, performed on 15 males between the ages of 65 years and 80 years, researchers measured postural balance responsiveness and simple reaction test (SRT) times in four testing phases: 1 week before Ramadhan, within the second week and the fourth week of Ramadhan, and 3 weeks after Ramadhan. The study found statistically significant impacts on postural balance and attentional capacities in the fasting subjects, mainly in the second week of Ramadhan, thereby increasing their risk for falls and fall-related injuries.^35^ These postural balance impairments were shown to require 3 weeks for recovery post-Ramadhan. An Indonesian study into the effect of Ramadhan fasting on the renal function of elderly subjects was recently undertaken.^26^ Results demonstrated lower eGFR in most subjects after Ramadhan fasting, compared to before or during Ramadhan, with statistically significant renal impairment noted in subjects with multiple co-morbidities.

From the religious perspective, Islamic scholars maintain that Ramadhan fasting is not mandatory upon the elderly with poor health status. The religion makes allowances for fasting-exemption through charitable acts, namely *fidyah,* where fasting is precluded by chronic disease and where the affected individual’s socio-economic status can provide for this. Despite this balanced spiritual provision, many elderly will insist upon observing the fast as their spiritual preference.

## Discussion

The high prevalence of diabetes, hypertension and associated renal impairment in the South African, and African context, requires that clinicians are aware of the potential impact that fasting can have on patients living with these conditions. Patient education should facilitate the risks of fasting, weighed against the potential spiritual and social benefits within the Islamic paradigm. This further requires that clinicians working with Muslim patients become sensitive to the psychosocial, cultural and spiritual value that this practice carries in their patients’ lives. While the literature indicates clear risk categories for diabetic patients, this is not the same for those patients with hypertension and renal disease, and the clinician would need to exercise their discretion on a case-by-case basis.

When counselling patients with cancer in relation to their risk associated with fasting, the experience, discretion and cultural sensitivity of the clinician need to foreground the approach to the situation. The literature does not indicate clear recommendations, but some valuable information is presented which assists clinicians in the counselling process. Probably the most important of the biomedical data relates to the need for good hydration and the maintenance of caloric and protein levels during the treatment and recovery phases. As such, an interdisciplinary approach to decision-making and counselling would be useful, specifically in consultation with nutritional and religio-cultural practitioners.

Ramadhan fasting impacts mental health variably, with early negative emotional states such as irritability and later positive psychological experiences such as a sense of reward, accomplishment, pride, and control. Further randomised controlled trials are required to strengthen these results, especially in populations that have not yet been tested.

However, even in the absence of stronger evidence, clinicians must realise that Muslim patients may opt to fast and should anticipate the short and longer-term impact of this practice.

While Ramadhan fasting carries immense physical, psycho-social and spiritual value to Muslims generally, it may pose serious threats to physical and mental health in the elderly, especially those with co-morbidities and multiple treatment regimens. For healthy elderly, fasting may pose minimal risk. However, even in the absence of chronic disease, the impact of fasting on hydration and postural control should be considered when discussing an individualised risk-benefit analysis with one’s patients. Shared decision-making, perhaps including the elder’s family or caregivers, may provide for a safe balance between patient autonomy and clinical safety-netting.

## Conclusion

Most of the literature reviewed on Ramadhan fasting is observational, with the possibility of implementing randomised controlled trials in this context being methodologically complex. However, systematic reviews were presented, with some being able to complete meta-analyses. The impact of Ramadhan fasting is on physical and mental health, while the social and spiritual factors also strongly influence patient behaviour. There is a dearth of local literature on the subject. Future research should explore the health-related experiences of local African Muslim populations, the impact of sociocultural factors on their decision-making, knowledge and attitudes of clinicians to this phenomenon, and optimising disease-specific evidence-based guidelines.
